# Physical activity change across retirement by device measured work-related and commuting activity

**DOI:** 10.1093/eurpub/ckac130.154

**Published:** 2022-10-25

**Authors:** S Pasanen, JI Halonen, K Suorsa, T Leskinen, Y Kestens, B Thierry, J Pentti, J Vahtera, S Stenholm

**Affiliations:** 1 Public Health, University of Turku, Turku University Hospital, Turku, Finland; 2 Centre for Population Health Research, University of Turku, Turku University Hospital, Turku, Finland; 3 Department of Health Security, Finnish Institute for Health and Welfare, Helsinki, Finland; 4 Centre de Recherche en Santé Publique, Montreal, Canada; 5 Clinicum, University of Helsinki, Helsinki, Finland

## Abstract

**Background:**

Work-related and commuting physical activity before retirement may contribute to changes in physical activity and sedentary time after retirement, and the aim of this study was to examine these associations.

**Methods:**

Study population consisted of participants of the Finnish Retirement and Aging study (n = 119). Activity behaviour was measured with GPS and accelerometer devices. The participants provided 637 measurement days before and 557 days after retirement. Work-related physical activity was defined as physical activity accumulated at workplace. Commuting activity was dichotomised based on the speed of trips between home and workplace to active (<20 km/h) and passive (≥20 km/h) commute. Participants were divided into four groups: non-active workers and commuters, non-active workers but active commuters, active workers but non-active commuters, and active workers and commuters. Linear regression analysis with generalized estimating equations were used for statistical analysis.

**Results:**

The change in physical activity during retirement transition markedly varied by the activity group. Lower work-related activity was associated with an increase in light physical activity and a decrease in sedentary time. Conversely, higher work-related activity was associated with a decrease in light physical activity and an increase in sedentary time, except among those active workers who were active commuters. Particularly the active workers but non-active commuters increased their sedentary time (48 min, 95% Cl 20 to 76) and decreased their light physical activity (54 min, 95% Cl -80 to -29). No statistically significant changes in moderate-to-vigorous physical activity were observed.

**Conclusions:**

Our results suggest that work-related physical activity is associated with changes in physical activity behavior when retiring. Special attention should be targeted to active workers who are non-active commuters to maintain physical activity and decrease sedentary time after retirement.

**Key messages:**

- Lower work-related physical activity before retirement was associated with an increase in light physical activity and a decrease in sedentary time after retirement.
- Active workers but non-active commuters showed an increase in sedentary time and a decline in light physical activity after retirement, and might be a suitable group for physical activity promotion.

